# Supplementary material for: Tracking Lexical and Semantic Prediction Error Underlying the N400 Using Artificial Neural Network Models of Sentence Processing
Source: Neurobiol Lang (Camb). 2024 Apr 1;5(1):136–66. doi: 10.1162/nol_a_00134 (PMC11025650; doi:10.1162/nol_a_00134)
Supplement: Supplementary file 1 [file nol-5-1-136-s001.pdf]

A. Electrodes used for the N400 region of interest

Figure 1. Easycap M10 sensor array used for EEG data collection in this study. The sensors highlighted in red are those included in the N400 definition for analyses based on the cluster used by Frank et al. (2015).

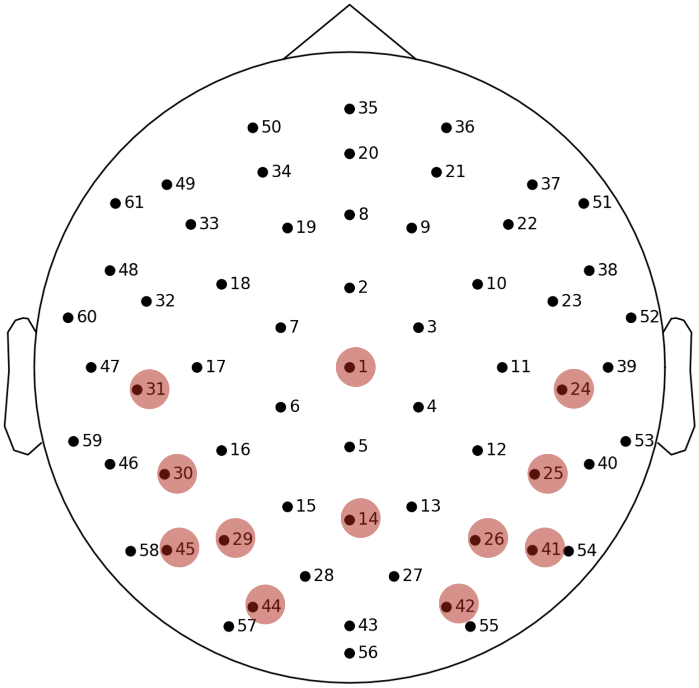

B. The Internal Update of the Language Model's Recurrent Layer

Here we assess the fit of the internal update of the recurrent layer of the LM (internal.LM) on the N400. This parallels the analyses conducted in the main text using SU.SGM, and as in those analyses we compare the effects of this regressor to surprisal estimated from the same LM (Section "Comparing surprisal and update as predictors of the N400"). In addition, we also compare the effect of internal.LM to SU.SGM.

Table 1. Linear mixed effect model fitted with the update of the LM (internal.LM) and aimed at predicting the amplitude of the N400 component.

	β	t	p
N400base	-0.41	-20.94	< 0.001
internal.LM	0.08	2.16	0.047

Table 2. Results of a model fitted with both surprisal and internal.LM and aimed at predicting the amplitude of the N400 component.

	β	t	p
N400base	-0.41	-21.00	< 0.001
internal.LM	0.10	2.94	0.004
surprisal.LM	0.17	3.20	0.003

Table 1 shows the results of a linear mixed effect model predicting the N400 as a function of internal.LM. The model included also the N400 baseline and was fit with per subject random slope and intercept and per word random intercept – internal.LM: $\beta = 0.08$, $t = 2.16$, $p = 0.047$ (FDR corrected). Table 2 contains the results of a linear mixed effect model fitted with internal.LM and surprisal.LM showing that both predictors derived from the language model are significant (surprisal.LM: $\beta = 0.17$, $t = 3.20$, $p = 0.003$; internal.LM: $\beta = 0.10$, $t = 2.94$, $p = 0.004$ FDR corrected).

We also measured the improvement in N400 prediction by incorporating internal.LM into a model already fitted with surprisal.LM, and vice versa. The models contains also the N400 baseline and were fitted with per-subject random slopes and random intercepts, along with per-word random intercepts. We conducted log-likelihood tests between the models reporting both χ^2 and ΔAIC.

Table 3. Results of analyses of variance and AIC reduction between a linear model of the N400 fit with lexical surprisal and a model fitted in addition with internal.LM (top row) and between a model of the N400 fit with internal.LM and a model fitted in addition with lexical surprisal.

**models**	χ^2	p	ΔAIC
surprisal.LM vs surprisal.LM+internal.LM	7.853	0.005	5
internal.LM vs surprisal.LM+internal.LM	9.225	0.002	7

In Table 3 we report the results of analyses of variance and AIC reduction between a linear model of the N400 fit with surprisal.LM and a model fitted with both surprisal.LM in addition with internal.LM ($\chi^2 = 7.853$, $p = 0.005$, $\Delta AIC = 5$), and between a model of the N400 fit with internal.LM and a

model fitted in addition with lexical surprisal ($\chi^2 = 9.225$, $p = 0.002$, $\Delta AIC = 7$).

Table 4. Results of a model fitted with both SU.SGM and internal.LM and aimed at predicting the amplitude of the N400 component.

	β	t	p
N400base	-0.41	-21.06	< 0.001
internal.LM	0.04	0.99	0.321
SU.SGM	0.20	6.19	< 0.001

Table 4 contains the results of a linear mixed effect model fitted with SU.SGM and internal.LM. The internal update of LM (internal.LM) fails to reach significance when SU.SGM is included in the model (SU.SGM: $\beta = 0.20$, $t = 6.19$, $p < 0.001$; internal.LM: $\beta = 0.04$, $t = 0.99$, $p = 3.21$ FDR corrected).

The improvement in N400 prediction was measured by incorporating internal.LM into a model already fitted with SU.SGM, and vice versa. The models contains also the N400 baseline and were fitted with per-subject random slopes and random intercepts, along with per-word random intercepts. We conducted log-likelihood tests between the models reporting both χ^2 and ΔAIC.

Table 5. Results of analyses of variance and AIC reduction between a linear model of the N400 fit with SU.SGM and a model fitted in addition with internal.LM (top row) and between a model of the N400 fit with internal.LM and a model fitted in addition with SU.SGM.

**models**	χ^2	p	ΔAIC
SU.SGM vs SU.SGM + internal.LM	0.9722	0.324	1
internal.LM vs internal.LM + SU.SGM	26.049	< 0.001	25

In Table 5 we report the results of analyses of variance and AIC reduction between a linear model of the N400 fit with SU.SGM and a model fitted with both SU.SGM in addition with internal.LM ($\chi^2 = 0.9722$, $p = 0.324$, $\Delta AIC = 1$), and between a model of the N400 fit with internal.LM and a model fitted in addition with SU:SGM ($\chi^2 = 26.049$, $p < 0.001$, $\Delta AIC = 25$).

In line with the main analyses, we replicated the same time-wise analyses on each separate electrode in the dataset and plotted the results in a series of topographical maps representing the distribution of the fit between internal.LM or surprisal.LM to electrophysiological activity using 50 ms wide non-overlapping time-windows.

Figure 2. (a) time-wise results of a series of independent linear mixed effect models predicting the EEG signal in a ROI defined over N400-sensitive electrodes as a function of the internal.LM (dark blue) and surprisal.LM (dashed blue); (b) topographical plot of the β-coefficients estimated by a linear mixed effect model predicting the EEG signal over time – from 0 to 700 ms post stimulus onset – as a function of **internal.LM** over 50 ms wide non-overlapping time-windows (thresholded at $p < 0.05$, FDR corrected).

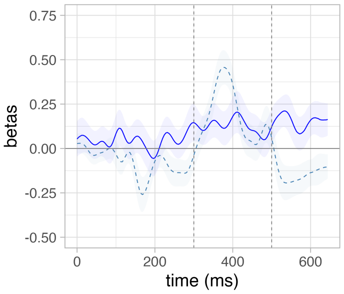

(a)

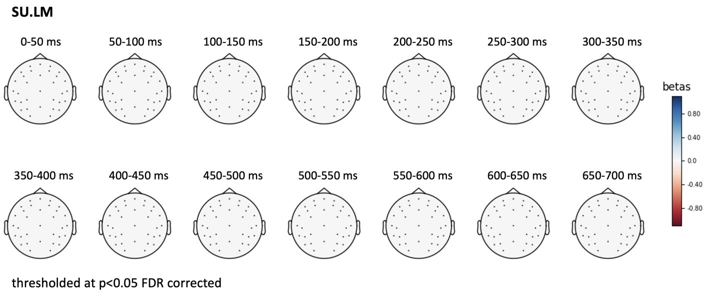

(b)

Figure 2a compares the time course of the effect of internal.LM (dark blue) and surprisal.LM (dashed blue) on the activity recorded in N400 sensitive electrodes as defined by (Frank et al., 2015). Figure 2b displays the topographical distribution the β-coefficients estimated by linear mixed effect models predicting EEG activity using internal.LM.

C. The effect of training

As humans acquire semantic knowledge about the world by experiencing their environment, the model learns knowledge about event semantics by being exposed to the statistic regularities in its environment – in this case the sentences contained in the RW-EN corpus – during training. In order to investigate whether training affects the similarity between the model's internal dynamics and the N400 amplitudes, we conducted similar analyses as in Section "Predicting the N400" using SU.SGM measures obtained from the same SG model before and after training.

Table 6 shows the results of a linear mixed effect models using as predictors of the N400 the SU.SGM computed from an untrained or a trained SG model. The effect of the SU.SGM of the untrained SG model is not significant ($\beta = -0.04$, $t = -1.63$, $p = 0.16$, FDR corrected).

Table 6. Comparison of the effects of the internal.LM estimated from an untrained (left) and trained (right) SG model.

	untrained			trained		
	β	t	p	β	t	p
N400base	-0.41	-20.86	< 0.001	-0.41	-21.01	< 0.001
SU.SGM	-0.04	-1.63	0.16	0.21	6.36	< 0.001

The effect of LM training on the relationship between surprisal and the amplitude of the N400 was evaluated using the same approach. Table 7 displays the outcomes of two linear mixed effect models that predicted the N400 using surprisal from either an untrained or a trained LM. The effect of surprisal from the untrained LM was not found to be significant ($\beta = -0.01$, $t = -0.50$, $p = 0.62$, FDR corrected).

Table 7. Comparison of the effects of surprisal.LM estimated from an untrained (left) and trained (right) LM.

	untrained			trained		
	β	t	p	β	t	p
N400base	-0.41	-20.86	< 0.001	-0.41	-21.02	< 0.001
surprisal.LM	-0.01	-0.50	0.62	0.15	2.79	0.008

These results seem to indicate that the models' ability to predict the N400 amplitude is strongly affected by the exposure to their training environment. It is evident from these results that the SU.SGM and surprisal obtained from a trained models better approximates the N400 as compared to the ones obtained from random models, i.e. models with randomly initialized connection weights.

Recently, Schrimpf et al. (2021) analyzed the fit between fMRI and ECoG data in the frontal-temporal cortex and vectorial representations generated by 43 deep learning models. Similarly to our study, besides using fully trained models, they also evaluate the same models before training. Interestingly, they observed that untrained networks yield representations that still significantly predict fMRI data, although training significantly improves fit. These results led to the proposal that the architecture of the networks can work as brain models of language even without extensive training because the structures implementing the deep learning networks might resemble similar neural mechanisms implemented by the cortical regions under analysis. We cannot conclude, based on our observations, that the architecture of the SG model alone – i.e. without training on a cognitively plausible task – is enough to approximate the electrophysiological processes under scrutiny. This partially diverges from Schrimpf et al. (2021)'s position. Nonetheless, we think it is important to stress that the difference between their and our conclusions might simply be due to the fact that the most successful models

in their study were implemented using architectures – such as the multi-head attention mechanism (Vaswani et al., 2017) – which were not used for the present iteration of the SG model. Moreover, instead of analyzing fMRI and ECoG data, we focused on EEG activity and in particular on the amplitude of the N400. Therefore, it could also be the case that our results simply reiterate the fact that the N400 ERP component's behavior evolves during an individual's experience of the statistical regularities of their environment, paralleled by the activity of the Sentence Gestalt layer during training epochs. Importantly, our results also emphasize that the implicit semantic prediction error reflected in N400 amplitudes inherently depends on the statistics of the environment as the predictions formed by the model (and presumably by human comprehenders) are generated based on the experience of these statistics.

Previously, Rabovsky et al. (2018) also investigated the effect of training on the SG model's ability to simulate N400 amplitudes, specifically during the processing of sentences containing semantically incongruent nouns. They reported that the SU.SGM shows at first an increase and later a decrease with additional training. These results are in line with the variation of the N400 during human language acquisition, which also first increases and then decreases across development (Atchley et al., 2006; Friedrich & Friederici, 2004; Kutas & Iragui, 1998). Moreover, Rabovsky et al. (2018) observed that the output layer activation approximates more and more the probability distributions embodied in the training corpus. This second point is in line with our results in confirming the role of training in improving the fit between a computational model and human data, mediated by the fit to the statistics of the world.

D. Controlling for surprisal from large-scale transformer models

In this section, we assess the fit of Semantic Update on the N400 together with surprisal estimated from GPT-2. In the main text, we compared the SU.SGM to the surprisal estimated from a LM with a comparable architecture that was trained on the same corpus (Section "Comparing surprisal and update as predictors of the N400"). This was because we felt that the disparity in number of parameters and training data between the SGM and state-of-the-art language models (such as GPT-2) could act as a confound in the assessment of the performance with regard to the N400 amplitude and would prevent a fair comparison between the mechanisms and hypotheses implemented by the models. However, we include this analysis for completeness.

We first fit a linear mixed effect model predicting the N400 as a function of both SU.SGM and surprisal estimated by GPT-2. The model included also the N400 base as described in the main text and per-subject random slopes and random intercepts, and per-word random intercepts.

Table 8. Linear mixed effect model fitted with the SG model SU (SU.SGM) and surprisal estimated by GPT-2 and aimed at predicting the amplitude of the N400 component.

	β	t	p
N400base	-0.41	-21.08	< 0.001
SU.SGM	0.10	3.05	0.003
surprisal.GPT2	0.36	8.73	< 0.001

Table 8 contains the results indicating that even with the presence of surprisal.GTP2 ($\beta = 0.36$, $t = 8.73$, $p < 0.001$ FDR corrected), SU.SGM makes a significant contribution to the amplitude of the N400 ($\beta = 0.10$, $t = 3.05$, $p = 0.003$ FDR corrected), and vice versa.

We also measured the improvement in N400 prediction by incorporating SU.SGM into a model already fitted with surprisal estimated by GPT-2, and vice versa. The models were fitted with per-subject random slopes and random intercepts, along with per-word random intercepts. We conducted a two log-likelihood test between the models reporting both χ^2 and ΔAIC.

Table 9. Results of analyses of variance and AIC reduction between a linear model of the N400 fit with surprisal estimated by GPT-2 and a model fitted in addition with SU.SGM.

**models**	χ^2	p	ΔAIC
surprisal.GPT2 vs SU.SGM + surprisal.GPT2	8.358	< 0.01	6
SU.SGM vs SU.SGM + surprisal.GPT2	39.372	< 0.001	37

Table 9 contains the results of two separate tests. The top row shows the comparison between a linear mixed effect model fitting surprisal and a model fitting both surprisal.GTP2 and SU.SGM. Adding SU.SGM to a model fitted with surprisal.GTP2 significantly improves it ($\chi^2 = 8.358$, $p < 0.01$ with $\Delta AIC = 6$). The bottom row instead compare a model fitting only SU.SGM to a model fitting both surprisal.GTP2 and SU.SGM, with results indicating a significance improvement after the introduction of surprisal ($\chi^2 = 39.372$, $p < 0.001$ with $\Delta AIC = 37$).

Furthermore, we replicated the same time-wise analyses on each separate electrode in the dataset and plotted the results in a series of topographical maps representing the distribution of the fit between SU.SGM or surprisal to electrophysiological activity using 50 ms wide non-overlapping time-windows. Figure 3a compares the time course of the effect of SU.SGM (dark red) and GPT-2 surprisal (dashed

green) on the activity recorded in N400 sensitive electrodes as defined by (Frank et al., 2015). Figure 3b displays the topographical distribution the β-coefficients estimated by linear mixed effect models predicting EEG activity using surprisal.GTP2.

Figure 3. (a) time-wise results of a series of independent linear mixed effect models predicting the EEG signal in a ROI defined over N400-sensitive electrodes as a function of the SU.SGM (red) and GPT-2 surprisal (green); (b) topographical plot of the goodness of fit of a linear mixed effect model predicting the EEG signal over time – from 0 to 700 ms post stimulus onset – as a function of **GPT-2 surprisal** over 50 ms wide non-overlapping time-windows (thresholded at $p < 0.05$, FDR corrected).

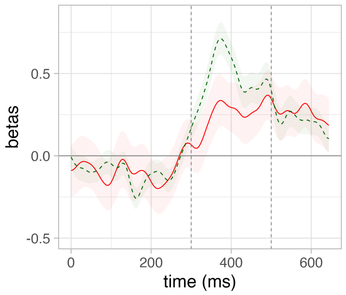

(a)

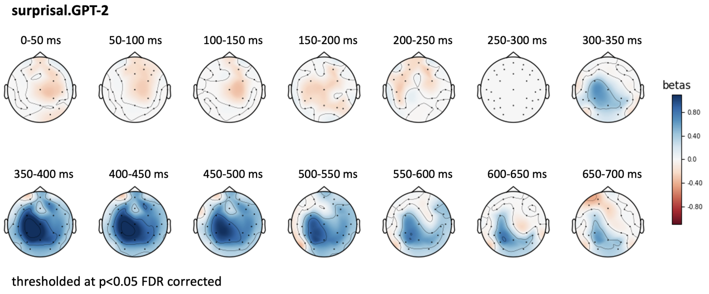

(b)

References

Atchley, R. A., Rice, M. L., Betz, S. K., Kwasny, K. M., Sereno, J. A., & Jongman, A. (2006). A comparison of semantic and syntactic event related potentials generated by children and adults. *Brain and Language*, *99*, 236–246.

Frank, S. L., Otten, L. J., Galli, G., & Vigliocco, G. (2015). The ERP response to the amount of information conveyed by words in sentences. *Brain and Language*, *140*, 1–11. https://doi.org/10.1016/j.bandl.2014.10.006

Friedrich, M., & Friederici, A. D. (2004). N400-like semantic incongruity effect in 19-month-olds: Processing known words in picture contexts. *Journal of Cognitive Neuroscience*, *16*, 1465–1477.

Kutas, M., & Iragui, V. J. (1998). The N400 in a semantic categorization task across 6 decades. *Electroencephalography and clinical neurophysiology*, *108 5*, 456–71.

Rabovsky, M., Hansen, S. S., & McClelland, J. L. (2018). Modelling the N400 brain potential as change in a probabilistic representation of meaning. *Nature Human Behaviour*, *2*, 693–705.

Schrimpf, M., Blank, I. A., Tuckute, G., Kauf, C., Hosseini, E. A., Kanwisher, N., Tenenbaum, J. B., & Fedorenko, E. (2021). The neural architecture of language: Integrative modeling converges on predictive processing. *Proceedings of the National Academy of Sciences*, *118*(45). https://doi.org/10.1073/pnas.2105646118

Vaswani, A., Shazeer, N., Parmar, N., Uszkoreit, J., Jones, L., Gomez, A. N., Kaiser, Ł., & Polosukhin, I. (2017). Attention is all you need. *Advances in Neural Information Processing Systems*, 5998–6008.
